# Comparative Genomic Analysis Reveals Habitat-Specific Genes and Regulatory Hubs within the Genus *Novosphingobium*

**DOI:** 10.1128/mSystems.00020-17

**Published:** 2017-05-23

**Authors:** Roshan Kumar, Helianthous Verma, Shazia Haider, Abhay Bajaj, Utkarsh Sood, Kalaiarasan Ponnusamy, Shekhar Nagar, Mallikarjun N. Shakarad, Ram Krishan Negi, Yogendra Singh, J. P. Khurana, Jack A. Gilbert, Rup Lal

**Affiliations:** a Department of Zoology, University of Delhi, Delhi, India; b School of Computational and Integrative Sciences, Jawaharlal Nehru University, New Delhi, India; c Synthetic Biology Laboratory, School of Biotechnology, Jawaharlal Nehru University, New Delhi, India; d Interdisciplinary Centre for Plant Genomics and Department of Plant Molecular Biology, University of Delhi South Campus, New Delhi, India; e The Microbiome Center, Argonne National Laboratory, Argonne, Illinois, USA; f Department of Surgery, The Microbiome Center, University of Chicago, Chicago, Illinois, USA; g The Microbiome Center, Marine Biological Laboratory, Woods Hole, Massachusetts, USA; Dalhousie University

**Keywords:** *Novosphingobium*, core genome, habitat-specific genes, pangenome, regulatory hubs

## Abstract

Species belonging to the genus *Novosphingobium* are found in many different habitats and have been identified as metabolically versatile. Through comparative genomic analysis, we identified habitat-specific genes and regulatory hubs that could determine habitat selection for *Novosphingobium* spp. Genomes from 27 *Novosphingobium* strains isolated from diverse habitats such as rhizosphere soil, plant surfaces, heavily contaminated soils, and marine and freshwater environments were analyzed. Genome size and coding potential were widely variable, differing significantly between habitats. Phylogenetic relationships between strains were less likely to describe functional genotype similarity than the habitat from which they were isolated. In this study, strains (19 out of 27) with a recorded habitat of isolation, and at least 3 representative strains per habitat, comprised four ecological groups—rhizosphere, contaminated soil, marine, and freshwater. Sulfur acquisition and metabolism were the only core genomic traits to differ significantly in proportion between these ecological groups; for example, alkane sulfonate (*ssuABCD*) assimilation was found exclusively in all of the rhizospheric isolates. When we examined osmolytic regulation in *Novosphingobium* spp. through ectoine biosynthesis, which was assumed to be marine habitat specific, we found that it was also present in isolates from contaminated soil, suggesting its relevance beyond the marine system. *Novosphingobium* strains were also found to harbor a wide variety of mono- and dioxygenases, responsible for the metabolism of several aromatic compounds, suggesting their potential to act as degraders of a variety of xenobiotic compounds. Protein-protein interaction analysis revealed β-barrel outer membrane proteins as habitat-specific hubs in each of the four habitats—freshwater (Saro_1868), marine water (PP1Y_AT17644), rhizosphere (PMI02_00367), and soil (V474_17210). These outer membrane proteins could play a key role in habitat demarcation and extend our understanding of the metabolic versatility of the *Novosphingobium* species.

**IMPORTANCE** This study highlights the significant role of a microorganism’s genetic repertoire in structuring the similarity between *Novosphingobium* strains. The results suggest that the phylogenetic relationships were mostly influenced by metabolic trait enrichment, which is possibly governed by the microenvironment of each microbe’s respective niche. Using core genome analysis, the enrichment of a certain set of genes specific to a particular habitat was determined, which provided insights on the influence of habitat on the distribution of metabolic traits in *Novosphingobium* strains. We also identified habitat-specific protein hubs, which suggested delineation of *Novosphingobium* strains based on their habitat. Examining the available genomes of ecologically diverse bacterial species and analyzing the habitat-specific genes are useful for understanding the distribution and evolution of functional and phylogenetic diversity in the genus *Novosphingobium*.

## Abstract (precis)

This study highlights the significant role of the genetic repertoire of a microorganism in the similarity between *Novosphingobium* strains. The results suggest that the phylogenetic relationships were mostly influenced by metabolic trait enrichment, which is possibly governed by the microenvironment of each microbe’s respective niche. Using core genome analysis, the enrichment of a certain set of genes specific to a particular habitat was determined, which provided insights on the influence of habitat on the distribution of metabolic traits for *Novosphingobium* strains. We also identified habitat-specific protein hubs, which suggested delineation of *Novosphingobium* strains based on their habitat. Examining the available genomes of ecologically diverse bacterial species and analyzing the habitat-specific genes are useful for understanding the distribution and evolution of functional and phylogenetic diversity in the genus *Novosphingobium*.

## INTRODUCTION

The genus* Novosphingobium* represents metabolically versatile members that belong to the class *Alphaproteobacteria* and family *Sphingomonadaceae* (1). *Novosphingobium* species have been isolated from a wide range of ecological habitats such as agricultural soil (2), pesticide-contaminated soil (3, 4), plant surfaces (5), and aquatic environments (6) (see Table 1). Previous studies have investigated *Novosphingobium* strains for their bioremediation capacity (7–10), nutrient cycling (11, 12), taxonomic characterization (3, 13), analysis of extracellular products (7), mutagenesis experiments on certain genes or gene clusters (14), disease conditions (15, 16), and application in nanoparticle formation for antibacterial activity (17).

Many *Novosphingobium* genomes are now available in public repositories (e.g., GenBank), and recently, Gan and colleagues (19) performed comparative genomic analysis where six *Novosphingobium* genomes were compared to elucidate the mechanism of salt tolerance, cell-cell signaling, and aromatic compound biodegradation. To further enhance our understanding of the metabolic versatility of this genus and to determine how this versatility is distributed by phylogeny and habitat, we selected 27 *Novosphingobium* genomes from diverse habitats and classified a subset of these strains into four different ecological groups—rhizosphere, contaminated soil, freshwater, and marine water. We then determined whether core metabolic trait distribution was influenced more by habitat or phylogenetic clustering.

## RESULTS AND DISCUSSION

### General genomic organization of *Novosphingobium* strains.

The 27 *Novosphingobium* strains had an average genome size of 4.97 Mbp. The largest genome was 6.95 Mbp, belonging to *Novosphingobium rosa* NBRC 15208 isolated from rhizospheric soil. The smallest genome was 3.71 Mbp, belonging to *N. acidiphillum* DSM19966, which was isolated from the acidic lake water. In order to investigate whether certain adaptive traits follow the environment-specific or habitat-specific phenotype, 27 *Novosphingobium* strains were grouped based on their isolation habitat. Of these 27 strains, 19 strains were grouped in one of the four different habitats, i.e., rhizosphere (strains AP12, P6W, and NBRC15208), contaminated soil (strains LL02, LE124, NBRC102051, KN65.2, and ST904), freshwater (strains AAP1, AAP83, AAP93, FNE08-7, DSM12444, and DSM19966), and marine water (strains MBES04, Musc273, DSM12447, US6-1, and PP1Y). The remaining eight strains (B-7, Leaf2, DSM13790, KF1, Rr2-17, NBRC 16725, NBRC 12533, and NBRC 107847) were excluded, as either there was no information available on their isolation site or less than three representatives were available to represent a habitat (Table 1). Focusing on the habitats, the largest genomes were found in the rhizosphere (6.37 ± 0.56 Mbp; *n* = 3), followed by contaminated soil (5.34 ± 0.55 Mbp; *n* = 5), marine water (5.21 ± 0.24 Mbp; *n* = 5), and freshwater (4.20 ± 0.34 Mbp; *n* = 6). Average genome size differed significantly between habitats (*F*_3,15_ = 16.89 and *P* < 0.0001 by analysis of variance [ANOVA]); it has previously been correlated with environmental complexity where the largest genomes are found in rhizospheric soil (18).

**TABLE 1:** General genome characteristic features of the genus* Novosphingobium*

Strain	Source of isolation	Genome size (bp)	No. of contigs/replicons^a^	GC content (%)	No. of genes	No. of essential marker genes	% completeness	Genomic island size (bp)	Accession no.	Reference
*Novosphingobium* sp. AAP1	Freshwater lake	4,750,579	50	65.6	4,304	106	99.07	501,818	LJHO00000000	Unpublished data
*Novosphingobium* sp. AAP83	Freshwater lake	4,232,088	84	59.4	4,074	106	99.07	286,348	LJHY00000000	Unpublished data
*Novosphingobium* sp. AAP93	Freshwater lake	4,267,112	149	65.5	3,948	104	97.20	219,491	LJHZ00000000	Unpublished data
*N. acidiphillum* DSM19966	Acidic lake water	3,708,535	55	64.3	3,496	104	97.20	248,334	AUBA00000000.1	Unpublished data
*N. aromaticivorans* DSM 12444	The sample obtained at a depth of 410 m from a borehole sample that was drilled at the Savannah River Site	4,233,314	One Chr and two plasmids	65.1	4,124	106	99.07	64,422	CP000248.1, CP000676.1, CP000677.1	Aylward et al. (12)
*N. fuchskuhlense* FNE08-7	Isolated from a surface water sample of the southwest basin of Lake Grosse Fuchskuhle	3,963,850	14	65.4	3,721	105	98.13	172,938	LLZS00000000.1	Unpublished data
*Novosphingobium* sp. MBES04	Sunken wood from Suruga Bay	5,361,448	33	65.4	5,202	103	96.26	528,404	BBNP00000000	Ohta et al. (5)
*N. malaysiense* Musc273	Mangrove sediment	5,027,021	85	63.4	4,887	106	99.07	135,248	JTDI00000000	Unpublished data
*N. pentaromaticivorans* US6-1	Muddy sediment of Ulsan Bay	5,457,578	One Chr and five plasmids	63.1	5,087	106	99.07	203,560	CP009291, CP009292, CP009293, CP009294, CP009295, CP009296	Choi et al., 2015 (81)
*Novosphingobium* sp. PP1Y	Marine water and oil interface	5,313,905	One Chr and three plasmids	63.3	5,135	106	99.07	181,419	FR856862.1, FR856859.1, FR856860.1, FR856861.1	D’Argenio et al. (6)
*N. subterraneum* DSM12447	Southeast coastal plain, subsurface core at 180-m depth	4,885,942	54	63.2	4,838	106	99.07	148,732	ZRVC00000000.1	Unpublished data
*Novosphingobium* sp. P6W	Isolated from the plant rhizosphere	6,537,300	65	63.7	6,279	105	98.13	322,771	JXZE00000000	Unpublished data
*Novosphingobium* sp. AP12	Rhizosphere of *Populus deltoides*	5,611,617	187	65.9	5,367	105	98.13	435,323	AKKE00000000	Unpublished data
*N. rosa* NBRC 15208	Isolated from root of plant *Rosa* sp. 3-ketolactose-forming bacteria	6,952,763	194	64.5	6,330	104	97.20	636,815	BCZE01000000	Unpublished data
*N. barchamii* LL02	Hexachlorocyclohexane-contaminated soil	5,307,348	26	64	5,220	104	97.20	264,580	JACU01000000	Pearce et al. (4)
*Novosphingobium* sp. KN65.2	Carbofuran-exposed agricultural soil	5,024,847	243	63.1	5,036	106	99.07	328,926	CCBH000000000	Nguyen et al. (2)
*N. lindaniclasticum* LE124	Hexachlorocyclohexane-contaminated soil	4,857,928	156	64.6	4,749	105	98.13	292,630	ATHL00000000	Saxena et al. (60)
*N. naphthalenivorans* NBRC102051	Isolated from polychlorinated-dioxin-contaminated environments	5,236,092	234	63.8	5,224	106	99.07	342,845	BCTX00000000.1	Unpublished data
*Novosphingobium* sp. ST904	Rhizosphere of *Acer pseudoplatanus*, growing at a 2,4,6-trinitrotoluene-contaminated forest site	6,269,463	166	64.5	6,945	100	93.46	303,084	LGJH00000000	Unpublished data
*Novosphingobium* sp. B-7	Steeping fluid of eroded bamboo slips	4,909,165	491	65.1	4,715	104	97.20	825,534	APCQ00000000	Unpublished data
*Novosphingobium* sp. Leaf2	Derived from an *Arabidopsis* leaf	3,715,735	22	64.1	3,675	106	99.07	235,969	LMJY00000000	Unpublished data
*N. nitrogenifigens* DSM 13790	New Zealand pulp mill effluent	4,148,048	77	64	3,867	106	99.07	255,167	AEWJ0000000	Unpublished data
*N. resinovorum* KF1	Biofilm of a bioreactor fed with polychlorinated phenols	6,304,486	115	65.1	6,079	106	99.07	279,955	JFYZ00000000.1	Unpublished data
*Novosphingobium* sp. Rr2-17	Grapevine crown gall tumor	4,539,029	166	62.7	4,513	104	97.20	295,587	AKFJ00000000	Gan et al., 2012 (19)
*N. tardaugens* NBRC 16725	Isolated from activated sludge of sewage treatment plant	4,291,514	54	61.3	4,223	105	98.13	319,713	BASZ00000000.1	Unpublished data
*N. capsulatum* NBRC12533	Not available	4,836,455	70	65.7	4,452	106	99.07	612,524	BCYV00000000.1	Unpublished data
*N. lentum* NBRC 107847	Isolated from a cold fluidized-bed process treating chlorophenol-contaminated groundwater	4,407,848	53	65.7	4,266	105	98.13	528,688	BCTW00000000.1	Unpublished data

a The number of contigs/replicons or the number of chromosomes (Chr) and plasmids is shown.

Previous studies based entirely on 16S rRNA gene sequencing predicted that the GC content in *Novosphingobium* varied between 62 and 67% (1, 12, 19). However, GC content of 27 *Novosphingobium* genomes in this study ranged from 59.4% in *Novosphingobium* sp. strain AAP83 to 65.9% in *Novosphingobium* sp. strain AP12. Based on essential marker gene analysis, the genomes of strain AP12 and AAP83 were >98% complete (Table 1); thus, the GC content range for the genus *Novosphingobium* as defined previously by DNA-DNA hybridization (DDH) should be reclassified to 59% to 67%. A previous study suggested that GC content is predicted to significantly influence the functional potential and hence ecological adaptation of an organism (20). However, the variability in percent GC content for *Novosphingobium* was not significant between the four habitats (*F*_3,15_ = 0.308 and *P* < 0.82 by ANOVA), suggesting that the ecological adaptations of *Novosphingobium* spp. are not influenced by a shift in percent GC content.

### Core genome and pangenome analysis.

Bacterial pangenomes typically consist of distinct core and accessory gene complements (21). *Novosphingobium* maintained a core gene complement of 220 genes (query coverage of ≥75% and nucleotide Identity of ≥75%) for the 27 genomes analyzed. As expected, these orthologs include components of regulatory pathways such as DNA replication, basic transcriptional machinery, translation, mismatch repair, nucleotide excision repair, homologous recombination, signal transduction, bacterial secretion system and protein export. In addition, citric acid cycle, fatty acid biosynthesis and elongation, amino acid biosynthesis and purine metabolism were also present. However, only 128 of the 220 orthologous genes could be reliably annotated as “essential” against the DEG database (22), whereas the remaining 92 accessory genes still coded for basic metabolic functions.

**FIG 1:**
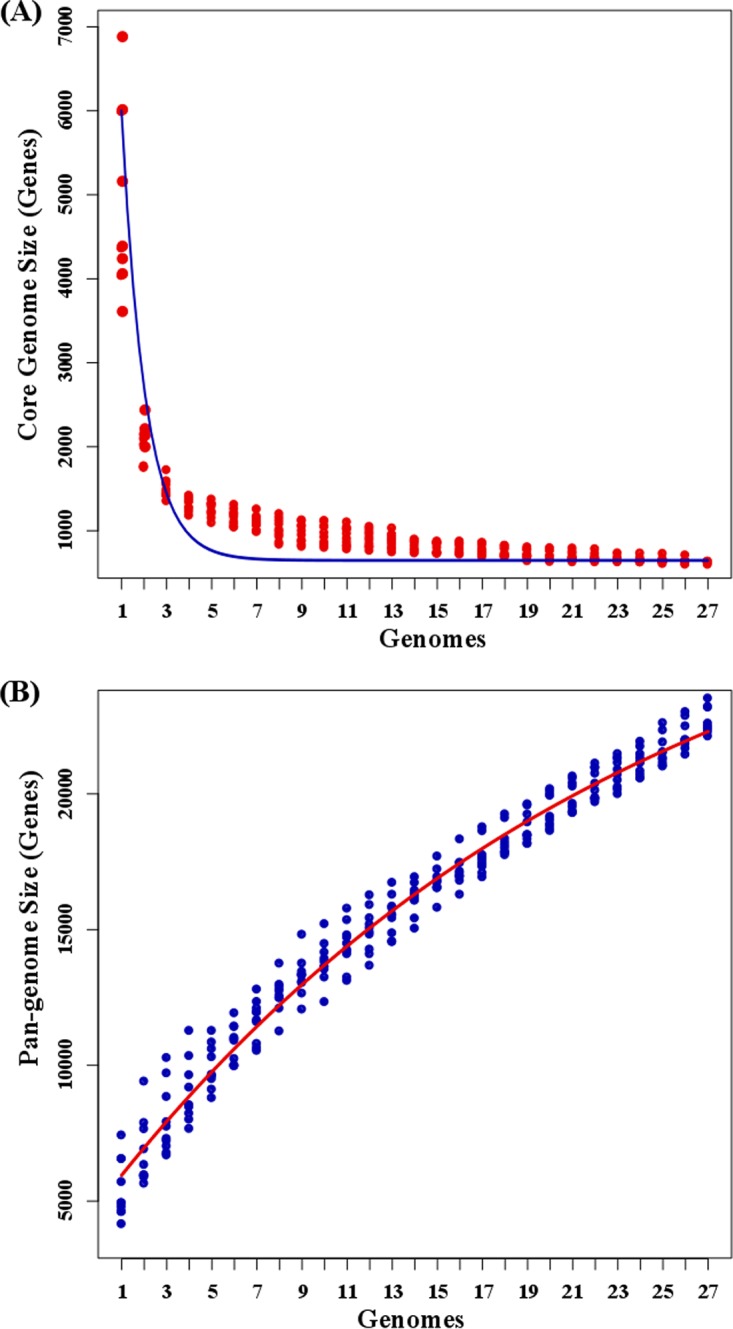
Core and pangenome of 27 *Novosphingobium* strains plotted against the number of genomes. (A) Core genome. The *x* axis shows the number of genomes, and the *y* axis shows the core genome size (number of genes) of *Novosphingobium* spp. (B) Pangenome. The *x* axis shows the number of genomes added, and the *y* axis shows the increase in pangenomic content of *Novosphingobium* spp. with the addition of genomes. The sizes of the core and pangenome clusters were computed using the BDBH algorithm. For the robustness of the calculation, the built-in program runs the sampling experiments (*n* = 10), where genomes are randomly added to estimate the stability of the core and pangenome. The best-fit Tettelin curve represents the regression line for the core and pangenome.

Pangenome analysis of the 27 *Novosphingobium* strains (Fig. 1) identified 21,915 nonredundant (nonrepetitive) genes in the pangenome, out of 128,647 total genes. The genome curve displayed an asymptotic trend, indicating that 27 genomes were insufficient to describe the complete gene repertoire of the genus *Novosphingobium*. Analysis of the core genome was also asymptotic, with 714 core genes after the addition of the 27th genome; however, this trend suggests that further *Novosphingobium* genomes will result in only minor changes in the core genome of this genus (Fig. 1).

### Habitat-specific traits.

The orthologous gene contents for *Novosphingobium* strains in four habitats were identified, and a pairwise comparison was performed to obtain habitat-specific genes. Out of 17,976 redundant orthologous genes, 1,943 gene sets were core genome for rhizosphere, 1,530 for contaminated soil, 1,485 for freshwater, and 1,546 for marine water. Further, comparison of the core genome of each habitat with respect to another revealed the presence of 438 specific genes for rhizosphere, 346 for contaminated soil, 143 for marine water, and 297 for freshwater. These habitat-specific genes were annotated against the KAAS server (23), but only 211 rhizospheric, 125 contaminated soil, 54 marine, and 150 freshwater genes could be annotated with a KEGG Orthology (KO) identifier. These KO identifiers were mapped against metabolic pathways using iPath (24), and the differences were mostly observed in amino acid metabolism, suggesting different amino acid availabilities in these environments (see Fig. S1 in the supplemental material). Rhizosphere-specific gene content consists of genes encoding components involved in glycine, serine, and threonine metabolism. Contaminated-soil-specific gene content consists of genes encoding components involved in tyrosine and phenylalanine metabolism. Freshwater-specific gene content contain genes encoding components involved in alanine, aspartate, and glutamate metabolism, and marine water-specific gene content contain genes encoding components involved in the bacterial chemotactic regulatory pathway, which could be involved in nutrient acquisition in this normally oligotrophic environment. Genes related to terpenoid backbone biosynthesis were present only in the core genomes of rhizospheric strains, which has been shown to play a role in the stability of bacterial cell membranes and root interaction in rhizospheric strains (25). Therefore, the analysis has put forward the differences between *Novosphingobium* strains based on differences in the metabolic preferences for amino acids in their respective habitats, representing the resultant adaptive changes in response to the environment.

10.1128/mSystems.00020-17.1 FIG S1  Mapping of habitat-specific metabolic pathways. The habitat-specific metabolic pathways were mapped using iPath v2. In the map, different habitats were represented by different colors: green for rhizosphere, yellow for contaminated soil, blue for marine water, and red for freshwater. Download FIG S1, TIF file, 8.3 MB. Copyright © 2017 Kumar et al. 2017 Kumar et al. This content is distributed under the terms of the Creative Commons Attribution 4.0 International license.

### Distribution of *Novosphingobium* strains along their phylogenetic clade.

**FIG 2:**
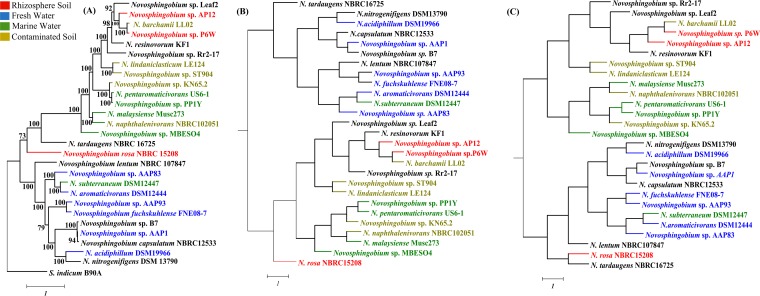
Phylogenetic clustering of 27 *Novosphingobium* strains. (A) Phylogeny based on 400 conserved marker genes with 1,000 bootstraps by using *S. indicum* B90A as an outgroup. (B and C) Average nucleotide identity (ANI)-based phylogeny was constructed with 220 orthologous genes and the whole genome, respectively. The bars represent 1 nucleotide substitution per position.

The consensus phylogeny of *Novosphingobium* spp. has shown the mixed trend of phylogenetic clustering of strains isolated from a similar environment. For instance, *N. barchamii* strain LL02 (contaminated soil), *Novosphingobium* sp. strain P6W (rhizosphere), and *Novosphingobium* sp. strain AP12 (rhizosphere), despite belonging to different environments, clustered together. While *Novosphingobium* sp. strain ST904 and *N. lindaniclasticum* LE124, which were both isolated from contaminated soil, form a monophyletic clade (Fig. 2 and Table 1). Notably, strains LL02 (13) and LE124 (3) were isolated from hexachlorocyclohexane (HCH) dumpsites, but in all three methods (conserved marker genes and average nucleotide identity [ANI] on the whole genome and core genome), these strains clustered separately. Similarly, *Novosphingobium* sp. strain KN65.2 was isolated from carbofuran-contaminated soil but clustered with marine isolates, *Novosphingobium* sp. strain PP1Y (6) and *N. pentaromaticivorans* US6-1 (10). This clustering is likely a result of shared metabolic tendency, as strain KN65.2 can degrade carbofuran (2) and strains PP1Y and US6-1 can degrade polyaromatic hydrocarbon (PAH) compounds (6, 10). Further ambiguity in habitat specificity was observed from the clustering of strains of marine, contaminated soil, and freshwater habitats (*N. malaysiense* Musc273 [marine], *N. naphthalenivorans* NBRC102051 [contaminated soil], *N. fuchskuhlense* FNE08-7 [freshwater], *Novosphingobium* sp. AAP93 [freshwater], *N. subterraneum* DSM12447 [marine], *N. aromaticivorans* DSM12444 [freshwater], and *Novosphingobium* sp. AAP83 [freshwater]). The results indicated that the phylogenetic clustering of genomes was apparently different from the habitat-specific grouping of these strains. This may be because *Novosphingobium* spp. have varied metabolic preferences, suggesting that habitat-specific factors are probably masked by the microenvironment in shaping the *Novosphingobium* genomes. Also, the differences in tree topology using these two methods, i.e., ANI (whole genome based) and 400 conserved bacterial marker genes, could be due to the inclusion of pangenomic content in the case of the whole genome (ANI) rather than the conserved marker genes. Further, to check the impact of the missing gene content from draft genomes, the phylogeny was constructed on the core genome using ANI. The result suggested that the least complete genome (≅93.46% [Table 1]), i.e., *Novosphingobium* sp. strain ST904 grouped with *N. lindaniclasticum* LE124 by all three methods. Thus, it can be inferred that the missing gene content will have the least impact on the change in phylogeny among the *Novosphingobium* strains.

### Habitat-specific protein identification and their protein-protein interaction analysis.

**FIG 3:**
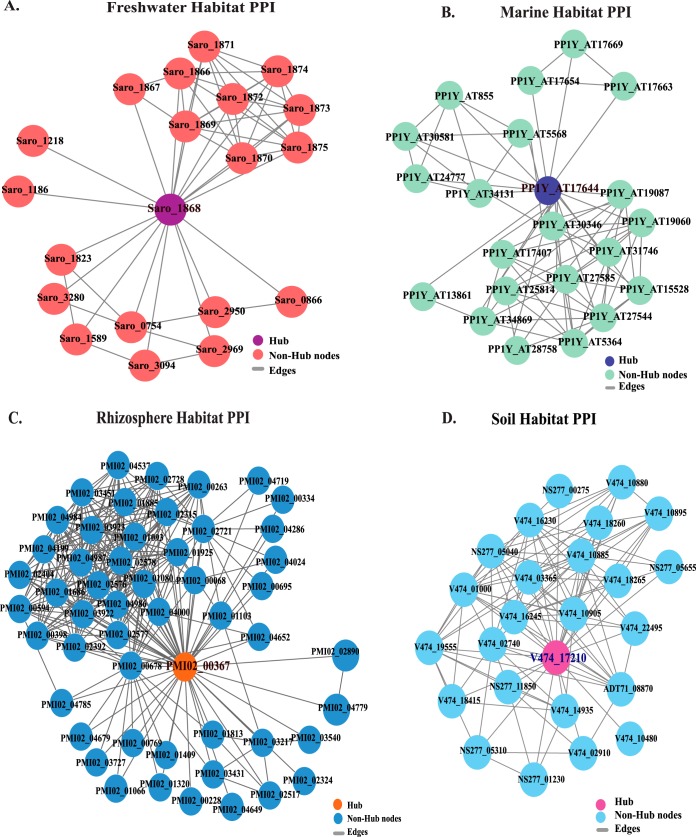
The protein-protein interaction (PPI) network of four habitats, i.e., freshwater, marine water, rhizosphere, and soil. Expanded view of the network imported from Cytoscape, where nodes represent proteins and edges represent physical interactions. The nodes in all four habitats (freshwater, marine water, rhizosphere, and contaminated soil) were represented as filled circles that were light red, green, dark blue, and light blue, respectively. The edges in all habitats were represented in the form of grey lines. The significant existence of sparsely distributed hubs in four habitat networks were represented by colored circles as purple (freshwater), dark blue (marine), orange (rhizosphere), and pink (contaminated soil).

The phylogenomics of the different strains did not reflect their habitat specificity, which suggests that the functional repertoire of these strains may supersede evolutionary relatedness. Protein-protein interaction (PPI) networks enable biological characteristics and protein function to be taken into consideration for each strain (26) and can be used to identify habitat-specific adaptations (27). To confirm that the proteome interaction with the environment, particularly for the uptake and secretion of molecules, is highly habitat specific, we aimed for the identification of putative outer membrane proteins involved in the transport of metabolites and toxins, as well as membrane biogenesis (28). We focused on proteins characterized as trans-membrane beta-barrel proteins (TMBbps) in *Novosphingobium* proteomes. The analysis showed the presence of different numbers of TMBbps in each strain of *Novosphingobium* across the four habitats. The identified TMBbp sequences of different strains clustered together based on habitat, when subjected to protein sequence similarity analysis. The proteins with the highest percentage of similarity were further referred to as habitat-specific proteins (HSPs). To validate their specificity toward the habitat, amino acid sequences of these TMBbps were subjected to phylogenetic analysis, which demonstrated habitat-specific clustering (Fig. S2). To confirm the stability of these proteins as key regulatory molecules, PPI interaction networks were established based on the core genome. To identify the key molecules, networks for each habitat were constructed and analyzed (Fig. 3A to D). The hub proteins for each strain in all four habitats were identified (Table S1). To understand the topological properties of these networks, the probability of degree distribution *P*(*k*) showed that each network followed a power law scaling behavior 

10.1128/mSystems.00020-17.2 FIG S2  Phylogenetic analysis based on habitat-specific beta-barrel protein using the neighbor-joining method and 1,000 bootstrap replicates. The evolutionary distances were computed by the Poisson correction method. Download FIG S2, EPS file, 1.5 MB. Copyright © 2017 Kumar et al. 2017 Kumar et al. This content is distributed under the terms of the Creative Commons Attribution 4.0 International license.

**FIG 4:**
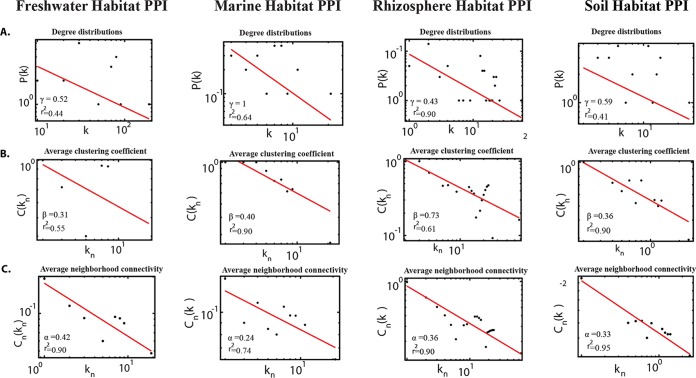
Topological properties of the PPI networks in the four habitats (freshwater, marine water, rhizosphere, and soil). The Pearson correlation coefficient values (*r*^2^) and probability of degree distributions *P*(*k*) (A), average clustering coefficient (B), and average neighborhood connectivity of the PPI network (C) are shown. All these properties follow the power law distribution and show the nature of scale-free network, suggesting a hierarchical organization in the network.

10.1128/mSystems.00020-17.3 TABLE S1  List of regulatory hub proteins in four different habitats of genus* Novosphingobium*. Download TABLE S1, DOCX file, 0.02 MB. Copyright © 2017 Kumar et al. 2017 Kumar et al. This content is distributed under the terms of the Creative Commons Attribution 4.0 International license.

(1) $$P(k)\sim k^{-\text{γ}}$$

with the values of the degree exponent γ were ∼0.52, 1.0, 0.43, and 0.59 in freshwater, marine water, rhizosphere, and contaminated soil habitats, respectively (Fig. 4A). The small value of γ (γ < 2) indicated that the network was hierarchical (29), signifying the emergence of hierarchical modules and/or communities (30), with a sparse distribution of highly connected hubs (31). The fact that these few highly connected hubs were connected to many low-degree nodes was indicative of a regulatory power of the hubs over these nodes. For further analysis of this topological feature of the network (30), the average clustering coefficient *C*(*k_n_*) was calculated as a function of the number of neighbors *k_n_*:

(2) $$C\;\left(k_{n}\right)\sim k_{n}^{-\text{β}}$$

Again, this followed the power scaling law with β values of ∼0.31, 0.40, 0.73, and 0.36 in freshwater, marine water, rhizosphere, and contaminated soil habitats, respectively, which supported that the network falls in a hierarchical network (Fig. 4B).

The average neighborhood connectivity *C*_*n*_(*k*_*n*_) was constructed as a function of *k*_*n*_ as follows:

(3) $$C_{n}(k_{n})\sim k_{n}^{-\text{α}}$$

with values of ~0.42, 0.24, 0.36, and 0.33 in freshwater, marine water, rhizosphere, and soil habitats, respectively (Fig. 4C), also indicating that the network falls in a hierarchical network (30, 31), the hub proteins in each habitat network are likely indicative of key molecules for habitat adaptation in each genome (32), and these proteins had the highest degree of interactions in these hierarchical networks. Hub proteins of each habitat were identified, and these proteins include the Saro_1868 protein (TonB-dependent receptor) for the freshwater habitat (Fig. 3A), PP1Y_AT17644 protein (hypothetical protein with porin domain) for the marine habitat (Fig. 3B), PMI02_00367 protein (TonB-dependent receptor) for the rhizosphere habitat (Fig. 3C), and V474_17210 protein (TonB-dependent receptor) for the soil habitat (Fig. 3D). As these β-barrel outer membrane proteins are present on the surfaces of Gram-negative bacteria and perform a variety of functions such as active ion transport, passive nutrient uptake, membrane anchors, membrane-bound enzymes, and in defense (33), they are likely crucial for the adaptation of the *Novosphingobium* strains in their respective environments.

### Sulfur uptake and metabolism are different between habitats.

**FIG 5:**
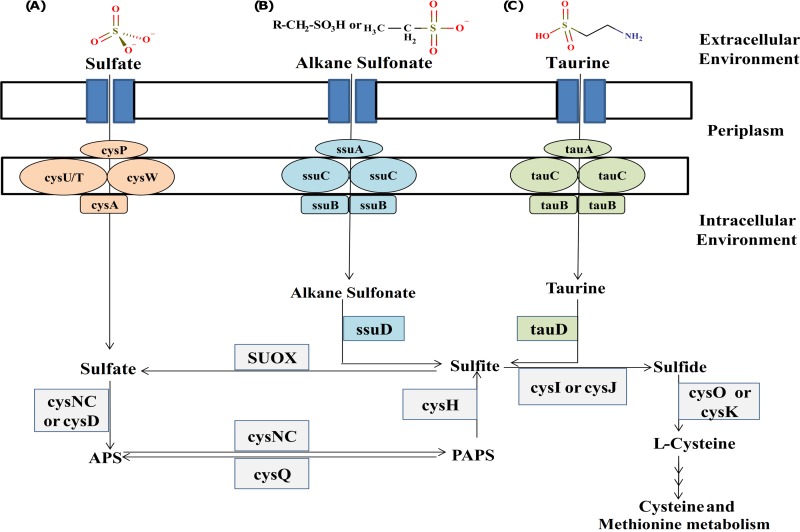
Schematic representation of different modes of environmental sulfur uptake and utilization within the *Novosphingobium* genus. The three different routes for sulfur assimilation are shown. Sulfur assimilation as inorganic sulfur (sulfates and thiosulfates) (A), via *ssuABC* (transport system) and *ssuD* (FMNH_2_-dependent alkane sulfonate monooxygenase) (B), and via taurine transport and metabolism by *tauABC* (transport system) and* tauD* (taurine dioxygenase) (C). APS, adenosine phosphosulfate; PAPS, phosphoadenosine phosphosulfate; SUOX, sulfite oxidase.

The sulfur metabolism pathway in prokaryotes involves the uptake and utilization of environmental sulfur derivatives for the synthesis of proteins, sulfate esters of polysaccharides, phenols, steroids, and coenzymes. In general, there are three different routes for the assimilation of environmental sulfur (Fig. 5). The first and predominant mode includes the uptake and metabolism of sulfates in the form of inorganic sulfur (sulfates and thiosulfates) which is carried out by proteins encoded by *cysPAUW* (transport system) (34) and *cysD* and *cysNC* (activation and utilization) (35) followed by cysteine biosynthesis genes *cysE*, *cysK*, and *cysQ*. The second route involves the uptake and utilization of environmental sulfonates, characterized by the presence of the *ssuABC* (transport system) and *ssuD* (FMNH_2_-dependent alkane sulfonate monooxygenase) genes. The alkane sulfonates comprise the major portion of carbon-bonded environmental sulfur (68%) (36) and 20 to 40% of organic sulfur present near marine sediments (37). The third route of sulfur assimilation involves taurine transport and metabolism encoded by the *tauABC* (transport system) and* tauD* (taurine dioxygenase) genes, respectively.

**FIG 6:**
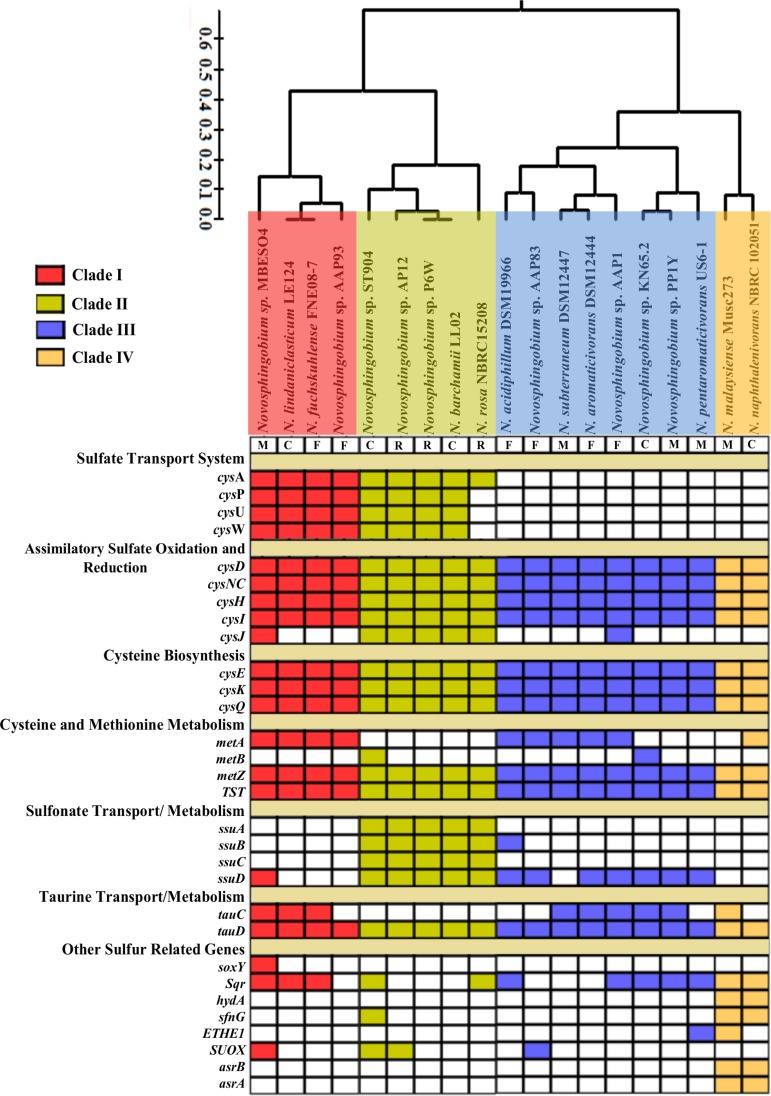
Matrix and dual dendrogram based on the presence/absence of sulfur metabolism genes was constructed in 19 *Novosphingobium* genomes belonging to four different habitats, viz., contaminated soil (C), rhizosphere (R), freshwater (F), and marine water (M). The colored and white boxes represent the presence and absence of a gene, respectively. A dendrogram based on the matrix of sulfur metabolism genes was constructed using Pearson correlation and hierarchical clustering.

Studies related to sulfur assimilation in bacteria isolated from different habitats have revealed the coexistence of these routes in the same species (38), but to date, no study has determined the distribution of these three pathways across different habitats. To determine this for *Novosphingobium* in rhizosphere, contaminated soil, freshwater, and marine water, the genes involved in sulfur metabolism were identified and strains were clustered according to their sulfur assimilation repertoire. Four resultant clades were designated: clade I, clade II, clade III, and clade IV (Fig. 6). Although clustering of the strains based on habitat was not observed, the pattern of differentiation of pathways was clearly demarcated. For instance, sulfate metabolism, the most predominant mode of environmental sulfur assimilation, was found only in clade I (strains MBES04, LE124, FNE08-7, and AAP93) and clade II (strains ST904, AP12, P6W, LL02, and NBRC15208) (Fig. 6). Further, the complete pathway of alkane sulfonate assimilation was found exclusively in strains clustered in clade II, which comprised only soil isolates (rhizosphere and contaminated soil). Earlier, the alkane sulfonate assimilation system had been reported in freshwater isolates (38), but none of the freshwater isolates we studied maintained the system. In addition to this, *tauD* coding for taurine dioxygenase was identified in all of the *Novosphingobium* strains, while the taurine transport system was absent. The two other clades, clades III (comprised of mainly aquatic isolates) and IV, lacked a complete sulfur transport system, instead maintaining a mosaic of genes encoding components involved in sulfate oxidation, taurine oxidation, and sulfonate oxidation, which suggests the use of multiple sulfur derivatives. Interestingly, the strains isolated from contaminated soil were found in all four clades and therefore maintained a diverse array of sulfate metabolism. This suggests that the modes of sulfur assimilation in *Novosphingobium* spp. were not confined to a certain habitat but might relate to the availability of different types of environmental sulfur compounds in their respective habitats.

### Mechanisms for survival in marine environments are also observed in contaminated soils.

In general, there are two different strategies that are known to confer bacterial survival in a saline environment. These strategies include accumulation of inorganic components in the cytoplasm, which counterbalances the salinity (39), and synthesis of the organic osmolytes that do not increase the ionic concentration but maintain the osmotic pressure (40). Two such osmotic solutes are ectoine (1,4,5,6-tetrahydro-2-methyl-4-pyrimidine carboxylic acid) and hydroxyectoine, which are common osmolytes in marine and halotrophic bacteria (41–43). The ectoine biosynthesis pathway involves components encoded by the *ectA* (L-2,4-diaminobutyric acid acetyltransferase), *ectB* (L-2,4-diaminobutyric acid transaminase), and *ectC* (l-ectoine synthase) genes (44). In addition to this, the protein encoded by *ectD* (ectoine hydroxylase) catalyzes the conversion of ectoine into hydroxyectoine (45).

Ectoine biosynthesis is considered to be an adaptation of marine *Novosphingobium* strains, such as *Novosphingobium* sp. strain PP1Y and *N. pentaromaticivorans* US6-1, which were previously reported to possess the ectoine biosynthesis pathway (19). However, we found that among the marine isolates, only *N. malaysiense* Musc273 along with PP1Y and US6-1 maintained a complete ectoine biosynthesis pathway, while two other marine isolates, *N. subterraneum* DSM12447 and *Novosphingobium* sp. strain MBES04, did not possess any of the ectoine pathway genes. The complete absence of the ectoine pathway in marine strains MBES04 and DSM12447 suggested that these strains might use different routes to compensate for high-salt conditions of marine water. Another possible reason might be that both strains are not truly marine, as the former was isolated from sunken wood (5) while the latter was isolated from coastal plains at a depth of 180 m (unpublished). Interestingly, strains isolated from other habitats were found to possess genes for ectoine biosynthesis, such as *Novosphingobium* sp. KN65.2, a carbofuran-contaminated soil isolate, which possessed the complete ectoine biosynthesis pathway. In addition to this, *ectA* and *ectB* were identified in *N. barchamii* LL02 and *Novosphingobium* sp. ST904, isolated from hexachlorocyclohexane- and 2,4,6-trinitrotoluene-contaminated soil, respectively. Also, rhizospheric strains, *Novosphingobium* sp. P6W and *N. rosa* NBRC 15208 were found to possess *ectA* and *ectB*, respectively, while freshwater strains were completely devoid of genes for ectoine biosynthesis. The occurrence of ectoine pathway genes in strains from contaminated soil and rhizosphere habitats implies that ectoine synthesis may not be a habitat-specific trait but it may instead be acquired and maintained by strains from different ecological niches, likely driven by environmental stress, or that the pathway is not useful but simply maintained in the contaminated soil environment.

### Degradation potential of *Novosphingobium* strains across four different habitats.

**FIG 7:**
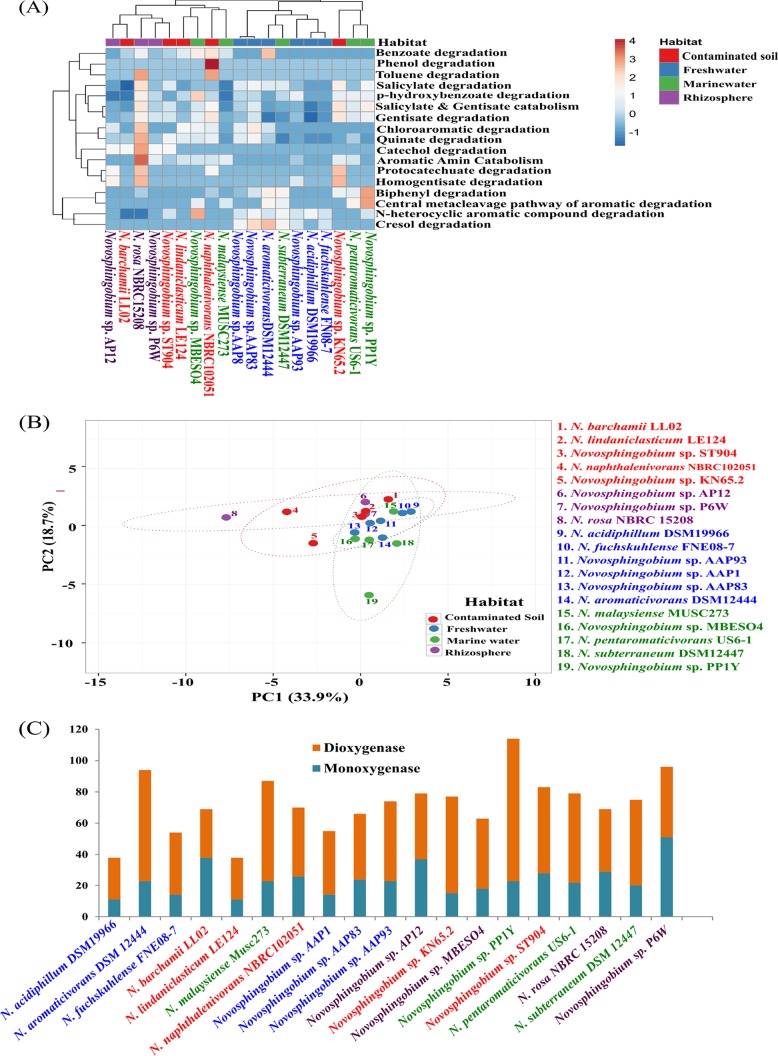
Correlation between the ability of *Novosphingobium* strains from four different habitats to degrade aromatic and xenobiotic compounds. (A) The heat map represents clustering of genomes based on the presence of different aromatic degradation pathways. (B) Principal-component analysis (PCA) plot using strain-specific degradation pathways. (C) Distribution of mono- and dioxygenase genes within *Novosphingobium* genomes.

Sphingomonads have been widely reported as efficient degraders of xenobiotic compounds such as hexachlorocyclohexane, chlorophenol, phenol, homogentisate, anthranilate, and other polyaromatic hydrocarbons (46, 47). Of the sphingomonads, *Sphingobium* and *Sphingomonas* strains have been extensively studied with respect to their xenobiotic degradation potential (12, 48), while less is known about *Novosphingobium* spp. A comparative genomic study on six *Novosphingobium* strains was carried out earlier (19), but the focus was on overall genomic repertoire. Here we analyzed *Novosphingobium* genomes for the presence of aromatic compound degradation pathway genes. The analysis revealed that the genes encoding PAH and components involved in xenobiotic degradation were enriched in *Novosphingobium* strains (Fig. 7) among which freshwater strains showed similarity in genes encoding mono- and dioxygenases, with very similar metabolic profiles, while strains from the other three habitats clustered separately (Fig. 7A and B). Of note, *N. rosa* NBRC15208, a rhizospheric isolate, was found to harbor the highest number of genes (*n* = 157) for aromatic compound degradation, especially for gentisate, protocatechuate, and catechol. The two other rhizospheric strains, *Novosphingobium* sp. P6W (*n* = 45) and *Novosphingobium* sp. AP12 (*n* = 59), contained only 33% of the *N. rosa* NBRC15208 gene complement. Following this, *Novosphingobium* sp. KN65.2 (contaminated soil) and *Novosphingobium* sp. PP1Y (marine) with 124 genes each, had the second highest metabolic repertoire. *Novosphingobium* sp. KN65.2 possessed genes mainly for gentisate, biphenyl, homogentisate, and protocatechuate degradation, while *Novosphingobium* sp. PP1Y possessed a high number of gentisate and biphenyl degradation genes. Interestingly, strains from sites contaminated with HCH, polychlorinated dioxin, pulp mill effluent, and carbofuran contained comparably fewer genes for aromatic compound degradation, which suggested that particular contaminants might lead to genome streamlining under environmental stress. Also, genes for gentisate, catechol, and protocatechuate catabolism were found in abundance, projecting their ability to degrade a variety of aromatic compounds (49).

The presence of mono- and dioxygenase family proteins in *Novosphingobium* spp. (50), i.e., enzymes known for aromatic ring cleavage, was also determined. *Novosphingobium* sp. PP1Y showed the greatest number of genes coding for mono- and dioxygenases (114 genes) (Fig. 7C). The most predominant types of monooxygenases in *Novosphingobium* strains include cyclohexanone monooxygenase, nitrotriacetate monooxygenase, vanillate monooxygenase, alkanal monooxygenase, toluene-4-monooxygenase, alkane sulfonate monooxygenase, and choline monooxygenases. Of these monooxygenases, the most abundant was alkane sulfonate monooxygenase (17 copies) in strain P6W. Major dioxygenases include alpha-ketoglutarate-dependent taurine dioxygenase, benzoate 1,2-dioxygenase, catechol 1,2-dioxygenase, catechol 2,3-dioxygenase, phenylpropionate dioxygenase, and protocatechuate 4,5-dioxygenase, while alpha-ketoglutarate-dependent taurine dioxygenases (15 copies) were the most abundant and observed in strain AP12. This high diversity of mono- and dioxygenases in *Novosphingobium* strains suggests their hidden potential to metabolize a wide variety of aromatic hydrocarbons. Also, the abundance of alkane sulfonate monooxygenases and taurine dioxygenases indicates an ability to utilize environmental sulfur via environmental alkane sulfonate and taurine, respectively.

### Phage integration and genomic adaptation.

Phage/virus integration in bacterial genomes is often considered a genomic adaptation mechanism of bacterial strains which enables novel gene acquisition and might be critical for survival. It has been reported that integrated prophages can constitute up to 20% of a bacterial genome (51), which eventually leads to strain emergence and diversification. Such genomic reservoirs have been shown to be highly diverse across aquatic and terrestrial ecosystems (52). In this study, 29 intact phages in *Novosphingobium* genomes (Table 2) were identified, with the greatest diversity in strains from contaminated soil (12 phages). Although most of the proteins encoded by the integrated phage were either phage related or hypothetical, a few of the annotated proteins, such as arsenic resistance, NADH-dependent flavin mononucleotide (FMN) reductases, dioxygenase, and permease, could provide improved resistance and degradation of polyaromatic hydrocarbons (PAHs) (Table 2).

**TABLE 2:** Characteristic features of predicted phages within the genus *Novosphingobium*

Strain	Putative *attL* or *attR* sequence	Region length (kbp)	% GC	Total no. of CDS^a^	No. of phage proteins	No. of hypothetical proteins	Presence (no.)/absence of genes encoding^b^:																					
							Integrase/transposes	Short-chain dehydrogenase/reductase SDR	Translation initiation factor IF-2	Transcription elongation factor NusA	CopG/Arc/MetJ/Ars family transcriptional regulator	Phage shock protein PspC	Methylase	Hsp33 protein	NADPH-dependent FMN reductase	LexA repressor	Putative lipoprotein	5-Oxoprolinase	OmpA/MotB	Nuclease	Plasmid pRiA4b ORF-3 family protein	Serine *o*-acetyltransferase	ATPase subunit C	Dioxygenase	Protein-tyrosine-phosphatase	Arsenical resistance	Membrane dipeptidase	Amino acid permease
*Novosphingobium* sp. KN65.2	GCGCCTGATGCGC	57.2	61.40	63	31	32	−	−	−	−	−	−	−	−	−	−	−	−	−	−	−	−	−	−	−	−	−	−
	CTCCCGCTCCGCCA	39.2	62.16	55	33	21	−	−	−	−	−	−	1	−	−	−	−	−	−	−	−	−	−	−	−	−	−	−
	Unresolved	23.8	65.32	30	20	8	−	−	−	−	−	−	−	1	1	−	−	−	−	−	−	−	−	−	−	−	−	−
	Unresolved	12	63.81	17	13	4	−	−	−	−	−	−	−	−	−	−	−	−	−	−	−	−	−	−	−	−	−	−
*N. barchamii* LL02	CAAGGCAGGGAA	34.9	63.00	49	22	25	−	−	−	−	−	−	−	−	−	1	1	−	−	−	−	−	−	−	−	−	−	−
	Unresolved	16.8	69.11	20	11	8	−	−	−	−	−	−	−	−	1	−	−	−	−	−	−	−	−	−	−	−	−	−
*N. lindaniclasticum* LE124	TGCGCGGCGCCTT	35.2	63.73	48	30	18	−	−	−	−	−	−	−	−	−	−	−	−	−	−	−	−	−	−	−	−	−	−
*Novosphingobium* sp. ST904	Unresolved	16.7	68.88	24	13	10	−	−	−	−	−	−	−	−	1	−	−	−	−	−	−	−	−	−	−	−	−	−
	GGGCGGTTAGCTCAGTTGGTAGAGCATCTCGTTTACAC	40.3	63.01	55	33	22	−	−	−	−	−	−	−	−	−	−	−	−	−	−	−	−	−	−	−	−	−	−
	GACGGCGCCGAGCAT	40.5	65.26	37	27	10	−	−	−	−	−	−	−	−	−	−	−	−	−	−	−	−	−	−	−	−	−	−
*N. napthalenivorans* NBRC102051	Unresolved	35.7	64.76	54	28	21	−	−	−	−	1	−	−	−	−	−	−	−	−	−	−	−	−	1	1	1	−	−
	TTCGGATCAGGCTCT	25.9	61.12	26	14	7	3	−	−	−	−	−	−	−	−	−	−	−	−	−	−	−	−	−	−	−	1	1
*Novosphingobium* sp. MBES04	GAGGGTGAGATG	36.1	61.61	27	13	9	2	−	−	−	−	−	−	−	−	−	−	2	−	−	−	−	−	−	−	−	−	−
	Unresolved	19.3	68.18	27	15	7	−	−	−	−	−	−	−	−	1	−	−	−	2	−	−	−	−	−	−	−	−	−
*Novosphingobium* sp. PP1Y	CGCCGCCGCTGGTCG	49.9	61.54	46	29	15	1	−	−	−	−	−	−	−	−	−	−	−	−	1	−	−	−	−	−	−	−	−
	Unresolved	18.5	63.16	24	14	5	3	−	−	−	1	−	−	−	−	−	−	−	−	−	1	−	−	−	−	−	−	−
*N. subterraneum* DSM12447	CCGACCAAAGCACGAACCCGCTCCGCGGGAGAGTCGCTTGGGGTGCCGTAGCGTAGTATTGTTCAGGCTTTGCGTGCGGC	24.6	62.74	31	14	12	−	−	1	1	1	1	−	−	−	−	−	−	−	−	−	−	−	−	−	−	−	−
*N. pentaromaticivorans* US6-1	Unresolved	23.3	63.07	29	24	5	−	−	−	−	−	−	−	−	−	−	−	−	−	−	−	−	−	−	−	−	−	−
*Novosphingobium* sp. P6W	AGGAGCCCACGC	35.3	62.47	43	29	14	−	−	−	−	−	−	−	−	−	−	−	−	−	−	−	−	−	−	−	−	−	−
*N. rosa* NBRC15208	Unresolved	23.8	64.75	31	23	8	−	−	−	−	−	−	−	−	−	−	−	−	−	−	−	−	−	−	−	−	−	−
*Novosphingobium* sp. AAP93	Unresolved	13.5	64.21	15	12	−	2	−	−	−	−	−	−	−	−	−	−	−	−	−	−	1	−	−	−	−	−	−
*N. fuschkulense* FNE08-7	Unresolved	30.9	62.27	43	28	15	−	−	−	−	−	−	−	−	−	−	−	−	−	−	−	−	−	−	−	−	−	−
*N. nitrogenifigens* DSM 13790	Unresolved	27.9	64.58	34	26	7	−	1	−	−	−	−	−	−	−	−	−	−	−	−	−	−	−	−	−	−	−	−
*N. resinovorum* KF1	Unresolved	19.6	68.73	25	15	9	−	−	−	−	1	−	−	−	−	−	−	−	−	−	−	−	−	−	−	−	−	−
*N. tardaugens* NBRC 16725	GATCAGCTTGCTATGGACAAGACAACCACACGGCC	23.5	59.62	23	13	9	1	−	−	−	−	−	−	−	−	−	−	−	−	−	−	−	−	−	−	−	−	−
*Novosphingobium* sp. Leaf2	CGGATTTTAAGTCCGCAGCGTCTACCATTCCGCCACGCCCGCAC	37.4	63.76	51	34	15	−	−	−	−	−	−	−	−	−	1	−	−	−	1	−	−	−	−	−	−	−	−
	Unresolved	25.1	66.40	29	19	8	−	−	−	−	−	−	−	1	−	−	−	−	−	−	−	−	1	−	−	−	−	−
*Novosphingobium* sp. Rr2-17	Unresolved	16.3	67.62	21	11	9	−	−	−	−	−	−	−	−	1	−	−	−	−	−	−	−	−	−	−	−	−	−
	TTGATGGCGACGC	52.3	60.80	37	27	10	−	−	−	−	−	−	−	−	−	−	−	−	−	−	−	−	−	−	−	−	−	−

a CDS, coding sequences.

b The presence or absence (−) of genes encoding the indicated protein or characteristic is shown. If the gene is present, the number of genes is shown.

*Novosphingobium* strains from marine habitats had the second greatest abundance of phage content. This may be due to the fact that viruses are very common in oligotrophic marine environments (53, 54). Interestingly, *Novosphingobium* sp. MBES04 acquired the gene encoding 5-oxoprolinase via phage-mediated horizontal gene transfer, which catalyzes 5-oxoproline conversion into glutamate. Pyroglutamic acid or 5-oxoproline is an osmolyte that helps in the maintenance of osmotic balance along with sucrose and ectoine, predominantly characterized in bacteria inhabiting environments with high salt concentrations (55). Further, studies have also shown the role of glutamate in osmoregulation (56). Although the complete pathway for pyroglutamic acid synthesis was absent, the strain MBES04 might be using an alternative pathway and thus acquiring these features for streamlining the genome with respect to the habitat. Apart from this, marine strains have shown the acquisition of *ompA* and *motB* genes (MBES04), generally found in the outer membranes of Gram-negative bacteria (57) and known to influence bacterial attachment (58). Hence, this is predicted to further boost the chemotactic behavior of marine bacteria. Further, the acquisition of phage-mediated transcription initiation factor, elongation factor, and regulators may help in activation of adaptive genes (59). Thus, *Novosphingobium* strains have shown the well-developed phage acquisition-adaptation machinery that might play an important role in combating stress from the environment they inhabit.

### Conclusions.

The phylogenetic relationship among *Novosphingobium* strains was not completely concordant with habitat, as only some strains clustered with strains from similar habitats. The overall genetic repertoire played a significant role in structuring the similarity between strains, suggesting that habitat has little influence on the phylogenetic relationship. However, a systems biology approach revealed habitat-specific protein hubs that were able to delineate *Novosphingobium* strains based on their habitats. Further, metabolic genes with significant habitat-specific delineation were determined. For instance, sulfur acquisition was differentially encoded among strains and habitats, while the alkane sulfonate assimilation pathway was common among all rhizospheric strains. The ectoine biosynthesis pathway, predominantly identified for osmoregulation in marine bacteria, was also identified in strains isolated from other habitats, suggesting its significance beyond the marine habitat. Aromatic compound degradation and abundance of mono- and dioxygenase genes across all strains in all habitats suggest that *Novosphingobium* represents an untapped resource for the field of biotechnology. Abundance of integrated phage and resultant acquisition of genes that confer stability in their habitat are signs of well-developed phage gene acquisition machinery in *Novosphingobium*.

## MATERIALS AND METHODS

### Gene prediction and annotation.

*Novosphingobium* genomes, including both draft and complete genomes, were retrieved from the NCBI database (Table 1). One strain, *Novosphingobium lindaniclasticum* LE124, was sequenced by our laboratory using an Illumina genome analyzer and 454 GS FLX titanium platform, and reads were assembled into 156 contigs (60). For all of the *Novosphingobium* strains, genome annotations were carried out using RAST version 2.0 (61) and gene caller Glimmer-3 (62). Orthologs were predicted using the sequence clustering algorithm, COGtriangles (63) available in GET_HOMOLOGUES software package (64) with both identity and query coverage of ≥75%, using amino acid sequences. Further, the presence of essential genes in the orthologs was identified using the Database of Essential Genes (DEG) version 13.3 (22). The genome completeness was estimated by analyzing the presence of 107 essential copy genes using the Comprehensive Microbial Resource as a database, where 107 hidden Markov models (HMMs) of essential copy genes were analyzed in all of the *Novosphingobium* strains (65).

### Pangenomic and core genomic trend analysis.

For each genome, amino acid sequences were retrieved from RAST version 2.0 (61) and were used for pangenome and core genome trend analysis using the bidirectional best-hits (BDBH) clustering algorithm (64) at default parameters. Thereafter, the number of genes was plotted against the number of genomes added in the analysis, with Tettelin fitted curve (82).

### Phylogenetic analysis.

In order to obtain congruency in the phylogeny of* Novosphingobium* strains, three different methods were used. In the first method, phylogenetic clustering was performed based on protein sequences of 400 marker genes of *Novosphingobium* strains (66). The maximum likelihood methodology was used for the construction of the phylogenetic tree, using *S. indicum* B90A as an outgroup. In order to further demarcate the phylogeny of *Novosphingobium* strains, two other methods based on pairwise average nucleotide identity (ANI) (67) were used; the first method involves pairwise ANI comparison between 220 orthologous genes, and the second method employed whole-genome sequences to account for both core and accessory genome content. Two-way matrices were prepared, and dendrograms were constructed by the Pearson correlation method and hierarchical clustering using MeV (68).

### Habitat-specific genes and their metabolic pathways.

In order to identify the habitat-specific traits of the genus *Novosphingobium*, we divided the genomes into four different habitats, rhizosphere, contaminated soil, marine water, and freshwater (Table 1). Strains belonging to these habitats were included for further analysis. The strains isolated from the rhizosphere were *Novosphingobium* sp. AP12, *Novosphingobium* sp. P6W, and *N. rosa* NBRC 15208. The strains isolated from contaminated soil were *N. barchamii* LL02, *N. lindaniclasticum* LE124, *N. naphthalenivorans* NBRC102051, *Novosphingobium* sp. KN65.2, and *Novosphingobium* sp. ST904. The strains isolated from freshwater were *Novosphingobium* sp. AAP1, *Novosphingobium* sp. AAP83, *Novosphingobium* sp. AAP93, *N. fuchskuhlense* FNE08-7, *N. aromaticivorans* DSM12444, and *N. acidiphillum* DSM19966. The strains isolated from marine water were *Novosphingobium* sp. MBES04, *N. malaysiense* Musc273, *N. subterraneum* DSM12447, *N. pentaromaticivorans* US6-1, and *Novosphingobium* sp. PP1Y. Initially, the core genome content of each habitat was predicted by clustering the genomes with the COGtriangles algorithm (as described above). Then, the core genome of each habitat was compared to identify the cloud content (i.e., genes that were present in ≤2 habitats). Further, habitat-specific genes were retrieved manually, mapped for metabolic pathways using KAAS (23), and visualized using iPATH version 2 (24).

### Identification of habitat-specific proteins and their protein-protein interactions.

To identify habitat-specific proteins (HSPs), the trans-membrane beta-barrel proteins (TMBbps) (28) were predicted based on the BOMP (Beta-barrel Outer Membrane protein Predictor) program (69). Then, protein sequences of all strains were subjected to TMBbp prediction, and potential proteins were selected for further analysis. All TMBbp sequences of each habitat group were compared using BLASTp, so that the similar proteins could be used for hub identification (70). The TMBbp sequence comparison identified similar sequences present in all of the strains from these four habitats. The topmost sequence is considered a habitat-specific protein (HSP) and subjected to validation using phylogenetic analysis. In order to construct the protein-protein interactions (PPIs), HSP sequences of *Novosphingobium* strains were searched against the STRING Database (v10) (71). Strains from freshwater and marine water habitats were searched against *Novosphingobium aromaticivorans* and *Novosphingobium* sp. strain PPIY, respectively, while the soil and rhizosphere strains sequences were queried against *Novosphingobium nitrogenifigens*. The STRING v10 database consisted of known and predicted PPIs, which included both direct (physical) and indirect (functional) associations. The associations were integrated with different sources such as genomic context, high-throughput experimental data, database and literature mining, and analysis of coexpressed genes. This allowed an agile exploration of the interactome network and included certain calculated parameters that weighed the reliability of a given interaction (i.e., the “edges” of the interactome network) between two proteins and also qualified the functional environment around any given protein and their interacting partners (i.e., the “nodes” of the interactome network) (72). The PPI networks were visualized using Cytoscape version 3.0.1 (73). The hubs are proteins having a high degree of interactions, randomly placed in the network, and have important functional roles. In the current study, the hubs were identified using network analyzer and Perl programming version 5.18.2.2.

### Statistical analysis of the network.

The statistical and functional significance of the network was measured using various statistical parameters, namely, probability of degree distribution, average clustering coefficient, and average neighborhood connectivity (74). The degree of probability distribution, *P*(*k*), of a network deﬁned by *P*(*k*) = ^*n*^*k/N*, which is the ratio of the number of nodes having a *k* degree in the network (^*n*^*k*) to the size of the network (*N*), was used to capture the network structure, identification of hubs, and modular organization of the network. The network we constructed obeyed the power law, *P*(*k*) ∼ *k^−γ^*, indicating the scale-free nature of the network, where γ is an order parameter that identified the different topological structure of a scale-free network. The clustering coefficient *C*(*k*), which is deﬁned by

(4) C (k) =2E/[k (k − 1)]

is the ratio of the number of edges *E* of the node having a *k* degree with neighbors to the total possible number of such edges,

(5) [k (k − 1)]/2

which is a measure of the topological structure of the network (75). The average clustering coefficient *C*(*k*) identiﬁes overall organization of formation of clusters in the network. Similar to *P*(*k*), *C*(*k*) may depend on network size and characterizes various properties of the network: (i) for scale-free and random networks where *C*(*k*) is independent of *k*, *C*(*k*) ∼ constant, and (ii) for hierarchical networks where *C*(*k*) follows power law scaling behavior, *C*(*k*) ∼ *k*^β^ with β ∼ 1. The neighborhood connectivity of a node is the number of neighbors connected to it and characterizes the correlation pattern of connectivity of interacting nodes in the network. This connectivity correlation would be measured by deﬁning a conditional probability

(6) $$P\;(k\prime_{n}|k_{n})$$

which is the probability of making a link from a node having degree *k*_*n*_ to another node of degree *k*′_*n*_ (76). Then, the average neighborhood connectivity of nodes with connectivity *k*_*n*_ is given by

(7) $$C_{n}\;\left(k_{n}\right)\;=\;\sum_{k\prime_{n}}k\prime_{n}P\;\left(k\prime_{n}\text{|}k_{n}\right)\sim \;k_{n}{}^{-\propto }$$

(76) following a power law scaling behavior with α < 1 for most of the real networks (31, 77). If *C*_*n*_(*k*_*n*_) is an increasing function of *k*_*n*_ (for negative values of α), then the topology of the network shows assortive mixing (78) where nodes with a high number of edges per node (high-degree nodes) have affinity to connect to other high-degree nodes in the network. However, from equation 3 with positive values for α is the signature of the network having hierarchical structure, where low-degree nodes tend to connect high-degree hubs (78) and few high-degree hubs present in the network try to control the low-degree nodes.

### Phage and genomic island prediction.

Genomes were searched for phage content using the online server PHAST (79). The phage content was then analyzed for the presence of phage-related, hypothetical, and bacterial genes (Table 2). Further, genomic islands (GIs) were predicted using IslandViewer (80).
