# Supplementary material for: Comparative Genomic Analysis Reveals Habitat-Specific Genes and Regulatory Hubs within the Genus Novosphingobium
Source: mSystems. 2017 May 23;2(3):e00020-17. doi: 10.1128/mSystems.00020-17 (PMC5443232; doi:10.1128/mSystems.00020-17)
Supplement: TABLE S1 [file sys003172102st3.docx]

| **Habitat** | **Strains** | **Hubs Protein Ids** |
| --- | --- | --- |
| Freshwater | *Novosphingobium* sp. AAP1 | IP65_05950 |
|  | *Novosphingobium* sp. AAP93 | IP83_00425 |
|  | *Novosphingobium* sp. AAP83 | IP81_17615 |
|  | *Novosphingobium acidiphillum* DSM19966 | WP_051280685 |
|  | *Novosphingobium aromaticivorans* DSM12444 | Saro_1868 |
|  | *Novosphingobium fuchskuhlense* FNE08-7 | AQZ52_12220 |
| Marine | *Novosphingobium* sp. MBESO4 | MBENS4_2685 |
|  | *Novosphingobium malaysiense* Musc273 | LK12_03250 |
|  | *Novosphingobium subterrraneum* DSM12447 | NJ75_00886 |
|  | *Novosphingobium pentaromaticivorans* US6-1 | NSU_0876 |
|  | *Novosphingobium* sp. PP1Y | PP1Y_AT17644 |
| Rhizosphere | *Novosphingobium* sp. AP12 | PMI02_00367 |
|  | *Novosphingobium* sp. P6W | TQ38_02260 |
|  | *Novosphingobium rosa* NBRC15208 | WP_068082532 |
| Contaminated Soil | *Novosphingobium barchamii* LL02 | V474_17210 |
|  | *Novosphingobium lindaniclasticum* LE124 | L284_22525 |
|  | *Novosphingobium naphthalenivorans* NBRC102051 | WP_067734057 |
|  | *Novosphingobium* sp. KN65.2 | SPHV1_40006 |
|  | *Novosphingobium* sp. ST904 | ADT71_08150 |
